# Takotsubo Cardiomyopathy Caused by Gastritis Without Typical Stressors: A Case Report

**DOI:** 10.7759/cureus.73317

**Published:** 2024-11-09

**Authors:** Abdullah Abdullah, Simran Bains, Saryah Alhejazi

**Affiliations:** 1 General Medicine, Frimley Health Foundation Trust/Wexham Park Hospital, Slough, GBR; 2 Internal Medicine, Frimley Health Foundation Trust, Slough, GBR

**Keywords:** gastritis, : myocardial infarction with no obstructive coronary atherosclerosis, non-st segment elevation myocardial infarction (nstemi), takotsubo cardiomyopathy (tcm), tcm

## Abstract

Takotsubo cardiomyopathy (TCM) is characterized by transient left ventricular dysfunction in the absence of significant coronary artery disease. First described in Japan in the 1990s by Sato et al., this unique reversible cardiomyopathy typically occurs in post-menopausal women and is frequently triggered by physical or physiological stress. Despite numerous studies, the pathogenesis and etiology of TCM are incompletely understood. However, the overlap in the initial clinical presentation of TCM and acute coronary syndrome (ACS), the increasing incidence of TCM, particularly after the COVID-19 pandemic, and the comparable long-term mortality risk of TCM patients highlight the importance of a better understanding of this condition. Less common triggers, including gastrointestinal disturbances, have been associated with TCM, as will be discussed in this case report.

This case presents a 46-year-old female who was admitted to the hospital with epigastric pain and vomiting and later developed chest pain with elevated cardiac biomarkers and ECG changes. Following a diagnosis of suspected non-ST elevation myocardial infarction (NSTEMI), coronary angiography revealed unobstructed coronary arteries and magnetic resonance imaging confirmed TCM. This case highlights that TCM can be triggered by non-classical stressors such as prolonged gastrointestinal symptoms. Early recognition and appropriate management can lead to a favorable prognosis.

## Introduction

Takotsubo cardiomyopathy (TCM) is an increasingly recognized cardiac syndrome characterized by temporary left ventricular dysfunction, often presenting with symptoms and electrocardiographic findings that closely resemble acute coronary syndrome (ACS) but without the presence of obstructive coronary artery disease [1,2]. Originally described in Japan in the 1990s, TCM was initially associated with sudden emotional stress, such as grief or fear, particularly in postmenopausal women [3]. However, as clinical understanding of TCM has evolved, research now indicates that the syndrome can be triggered by a broader range of stressors, including physical and physiological stress from acute illnesses, infections, or even gastrointestinal disturbances [4]. 

The diagnosis of TCM remains challenging due to its overlap with ACS in both presentation and biomarker elevation, often leading to initial misdiagnosis. With an apparent increase in cases following the COVID-19 pandemic, likely due to heightened stress levels and health complications, TCM has become a condition of growing interest in cardiology [3]. This case report discusses an unusual presentation of TCM in a patient with chronic gastrointestinal symptoms, illustrating the importance of considering nontraditional triggers in the differential diagnosis. Understanding and identifying TCM early in its course is crucial for managing acute complications and ensuring appropriate patient outcomes.

## Case presentation

A 46-year-old female presented to the emergency department with a four-month history of constant, sharp epigastric pain, associated with continuous vomiting and multiple episodes of diarrhea. She denied hematemesis, melena, fever, shortness of breath, or neurological symptoms. She had a prior history of systemic lupus erythematosus (SLE) arthritis, which had been in remission for several years, and she was not on any current medications for SLE. The patient had sought medical attention twice for these symptoms, and a diagnosis of gastritis was made following a CT scan of the abdomen and pelvis and an esophagogastroduodenoscopy (OGD), both of which were unremarkable. Upon the current admission, the patient’s initial ECG showed a prolonged QT interval and biphasic T waves in lead V2. Serum electrolyte analysis revealed hypokalemia and hypomagnesemia, likely secondary to persistent vomiting; Table 1 shows blood results on admission.

**Table 1:** Blood result tests on admission Hb: Hemoglobin, WBC: White blood cells, MCV: Mean corpuscular volume, MCH: Mean corpuscular hemoglobin, MCHC: Mean corpuscular hemoglobin concentration, RDW: Red cell distribution width, MPV: Mean platelet volume, PT: Prothrombin time, APTT: Activated partial thromboplastin time, Na: Sodium, K: Potassium, Cr: Chromium, GFR: Glomerular filtration rate, ALP: Alkaline phosphatase, ALT: Alanine aminotransferase, Mg: Myasthenia gravis , CRP: C-reactive protein, LDL: Low-density lipoprotein, TSH: Thyroid stimulating hormone

Parameter	Result	Reference Range
Hb	102 (low)	130-170 g/L
WBC	11.6 (high)	4.0-11.0 x10^9/L
Platelets	221	150-400 x10^9/L
Hematocrit	0.314 (low)	0.36-0.50
MCV	79 (low)	80-100 fL
MCH	25.7 (low)	27-32 pg
MCHC	325 (low)	310-360 g/L
RDW	19.9 (high)	11.5-14.5%
MPV	11.4	7.5-11.5 fL
Neutrophils	8.9 (high)	2.0-7.5 x10^9/L
Lymphocytes	1.8	1.0-4.0 x10^9/L
Eosinophils	0.1	0.02-0.5 x10^9/L
Monocytes	0.8	0.2-0.8 x10^9/L
Basophils	0.1	0-0.2 x10^9/L
PT	13.7	11.5-13.5 s
PT Ratio	1.13	0.8-1.2
APTT	30.6	25-35 s
APTT Ratio	0.98	0.8-1.2
Vitamin B12	482	200-900 pg/mL
Folate	2.8 (low)	>3 ng/mL
Na	139	135-145 mmol/L
K	3.1 (low)	3.5-5.1 mmol/L
Cr	49	44-133 µmol/L
GFR	>90	>90 mL/min
Urea	3.1	2.5-7.1 mmol/L
Bilirubin	10	0-20 µmol/L
Albumin	42	35-50 g/L
ALP	79	30-130 U/L
ALT	20	<41 U/L
Mg	0.68 (low)	0.7-1.0 mmol/L
CRP	1.4	<5 mg/L
Cholesterol	3.8	<5 mmol/L
HDL Cholesterol	0.79 (low)	>1.0 mmol/L
Non-HDL	3	<3.0 mmol/L
Chol/HDL Ratio	4.8	<5
Triglycerides	2 (high)	<1.7 mmol/L
LDL	2.1	<3.0 mmol/L
TSH	2.39	0.27-4.20 mIU/L
Free T4	12.5	11.5-22.7 pmol/L
Free T3	3.5	3.5-6.5 pmol/L
Iron	8 (low)	10-30 µmol/L
Transferrin Saturation	10 (low)	20-50 %
Ferritin	18 (low)	30-300 µg/L
Transferrin	3.02	2.0-3.6 g/L
NT-proBNP	7516 (high)	<125 ng/L

**Figure 1:**
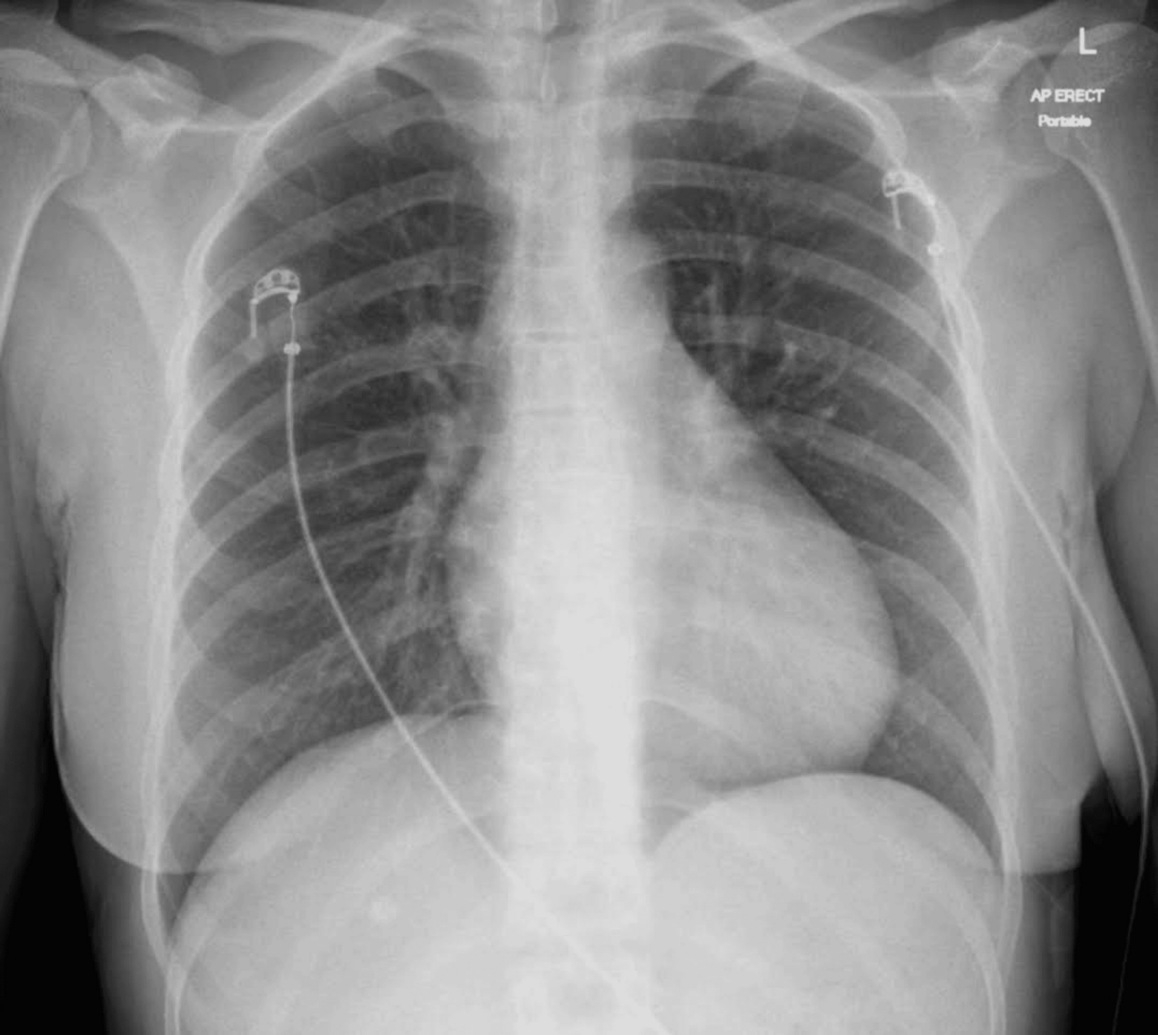
The chest X-ray on admission was showing clear lung fields, normal heart size, normal bony structure, and no pleural abnormalities.

She had a chest X-ray on initial admission, which did not show any abnormality; Figure 1 shows the CXR image.

**Figure 2:**
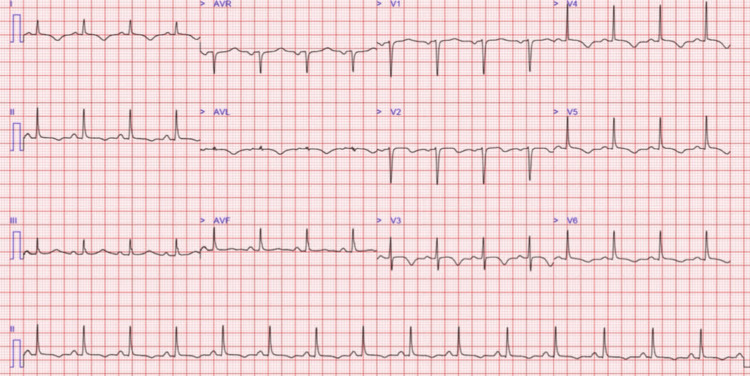
ECG when she had chest pain: T wave inversion was seen in lead 1, lead 2, avl, and all precordial leads v 1-6.

Two days into her hospitalization, she developed sudden, severe chest pain radiating to her back. Repeat ECG revealed dynamic changes, with new T-wave inversions across all precordial leads; Figure 2 shows the ECG while she had chest pain. Her troponin I level was elevated at 2114 ng/L (reference range: <0.04 ng/L), suggesting acute myocardial injury. She was subsequently treated as a case of non-ST elevation myocardial infarction (NSTEMI), and the ACS protocol was initiated, including dual antiplatelet therapy and fondaparinux [5].

Coronary angiography, however, revealed unobstructed coronary arteries, ruling out significant coronary artery disease. A contrast-enhanced CT aortogram was also performed to exclude aortic dissection, which was normal. Despite this, her chest pain persisted intermittently, and serial troponin levels showed a downward trend (2114 ng/L to 65 ng/L over three days). Brain natriuretic peptide (BNP) was elevated at 7500 pg/mL.

Given the lack of significant coronary artery disease and the dynamic ECG changes, Takotsubo cardiomyopathy was suspected. An echocardiogram showed a dilated left ventricle with severely impaired systolic function, with an ejection fraction (LVEF) of 29%. There was akinesis of the mid-to-apical walls, consistent with TCM [6].

**Figure 3:**
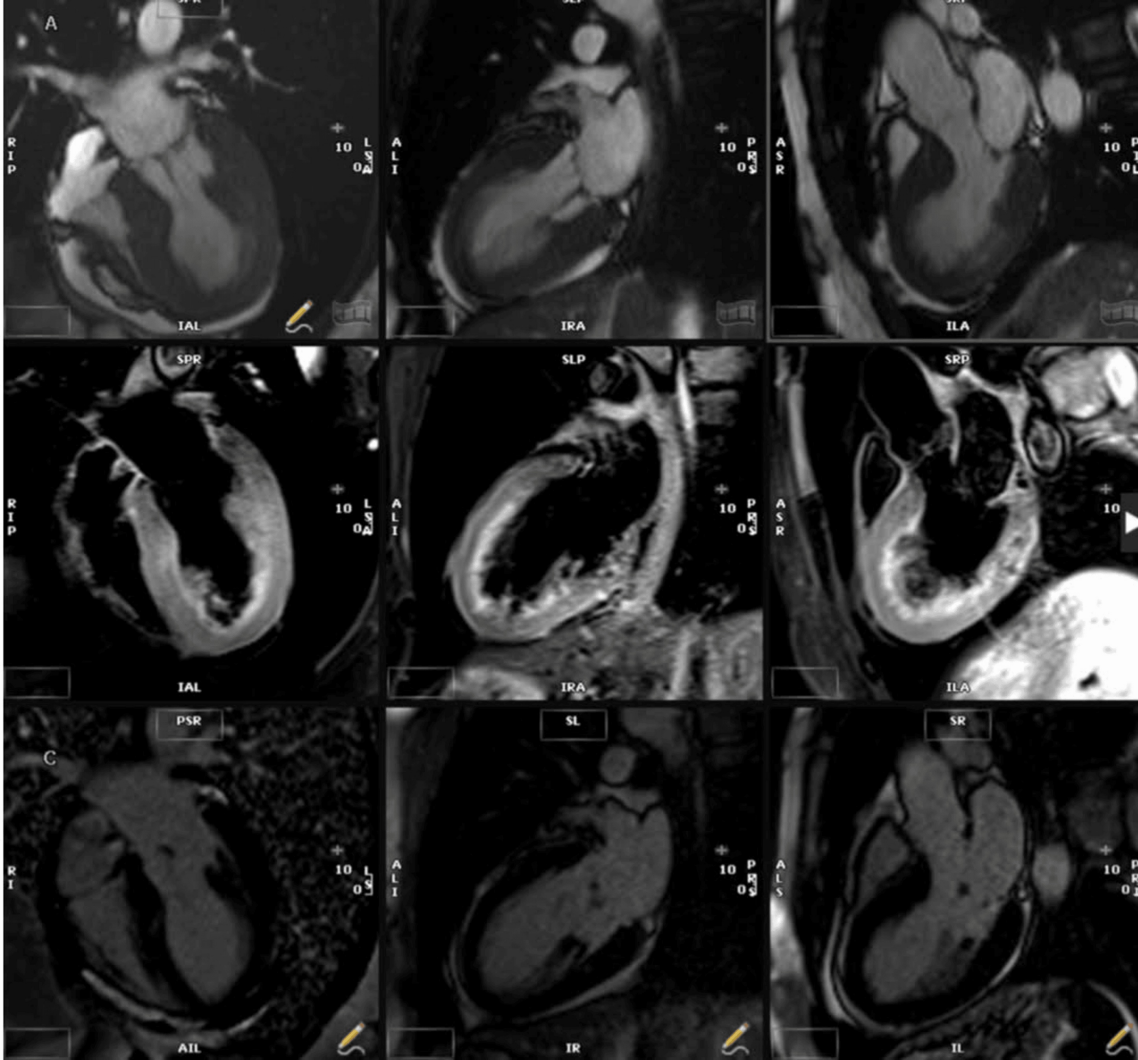
MRI showing features of TCM: myocardial edema was noted in the mid-to-apical segments, and patchy faint late gadolinium enhancement (LGE) was seen in the mid-septum, without any evidence of fibrosis or infarction.

Cardiac magnetic resonance imaging (MRI) was performed to confirm the diagnosis. MRI revealed a borderline severely impaired left ventricular systolic function with an LVEF of 37%, basal hyperkinesis, and apical akinesis. Diffuse myocardial edema was noted in the mid-to-apical segments, and patchy, faint late gadolinium enhancement (LGE) was seen in the mid-septum without any evidence of fibrosis or infarction [7]. These findings were entirely consistent with Takotsubo cardiomyopathy. Figure 3 shows the MRI images, which were suggestive of TCM features.

She was started on cardioprotective therapy, including beta-blockers and ACE inhibitors, to support left ventricular function. The patient's symptoms gradually improved, and she was discharged home with plans for close outpatient follow-up.

## Discussion

This case highlights an atypical presentation of Takotsubo cardiomyopathy, which in this instance was likely triggered by chronic gastrointestinal symptoms rather than a classical emotional or physical stressor. Traditionally, TCM is associated with intense emotional stress, such as grief, depression, or sudden shock [8]. However, recent studies have shown that up to one-third of cases are precipitated by non-emotional stressors, including medical illnesses like sepsis, hypoglycemia, or gastrointestinal disturbances like gastritis, pancreatitis, or cholecystitis [9]. In this case, the patient’s prolonged vomiting, abdominal pain, and electrolyte imbalances likely created a stressful physiological state, leading to the development of TCM.

The pathophysiology of TCM is believed to involve a surge of catecholamines, which induce myocardial stunning and dysfunction. This surge can be triggered by both emotional and physical stressors. Gastrointestinal disorders, such as gastritis, can cause significant physical distress, leading to the release of catecholamines [10]. This can explain how a relatively mild gastrointestinal condition, such as gastritis, could precipitate TCM in a susceptible individual.

The typical clinical presentation of TCM includes acute chest pain, dyspnea, and ECG changes that resemble ACS, such as ST-segment elevation or T-wave inversions [11]. However, the absence of obstructive coronary artery disease on angiography differentiates TCM from true ACS [12]. In this patient, the initial suspicion of NSTEMI was supported by elevated troponin levels and dynamic ECG changes, but the subsequent coronary 5 of 6 angiograms revealed normal arteries, prompting a reevaluation of the diagnosis. Imaging studies, particularly echocardiography and cardiac MRI, play a crucial role in confirming the diagnosis by demonstrating the characteristic wall motion abnormalities, typically affecting the apical and mid-ventricular segments [13].

This case also underscores the importance of recognizing electrolyte disturbances, such as hypokalemia and hypomagnesemia, as potential contributors to the development of TCM. These abnormalities, likely caused by the patient’s vomiting, not only contributed to her prolonged QT interval but may have also played a role in the myocardial dysfunction observed in TCM [4].

While the prognosis for TCM is generally favorable, with most patients experiencing full recovery of left ventricular function within weeks to months, complications such as heart failure, arrhythmias, and thromboembolism can occur in the acute phase [13]. Close follow-up is essential to ensure recovery and to monitor for potential long-term complications.

## Conclusions

Takotsubo cardiomyopathy is a condition that mimics ACS but is characterized by transient left ventricular dysfunction in the absence of significant coronary artery disease. This case demonstrates that TCM can be triggered by non-traditional stressors, such as prolonged gastrointestinal symptoms and vomiting, rather than emotional trauma or extreme physical stress. Awareness of such atypical presentations is crucial for timely diagnosis and management. Early use of imaging modalities, including echocardiography and cardiac MRI, can aid in differentiating TCM from other causes of acute chest pain. With appropriate treatment, most patients with TCM can expect a favorable outcome.

## References

[1] Prasad A, Lerman A, Rihal CS (2008). Apical ballooning syndrome (Takotsubo or stress cardiomyopathy): A mimic of acute myocardial infarction. Am Heart J.

[2] Templin C, Ghadri JR, Diekmann J (2015). Clinical features and outcomes of Takotsubo (stress) cardiomyopathy. N Engl J Med.

[3] Lyon AR, Rees PS, Prasad S (2008). Stress (Takotsubo) cardiomyopathy: A novel pathophysiological hypothesis to explain catecholamine-induced acute myocardial stunning. Nat Clin Pract Cardiovasc Med.

[4] Stiermaier T, Moeller C, Oehler K (2016). Long-term excess mortality in takotsubo cardiomyopathy: predictors, causes and clinical consequences. Eur J Heart Fail.

[5] Wittstein IS, Thiemann DR, Lima JA (2005). Neurohumoral features of myocardial stunning due to sudden emotional stress. N Engl J Med.

[6] Eitel I, von Knobelsdorff-Brenkenhoff F, Bernhardt P (2011). Clinical characteristics and cardiovascular magnetic resonance findings in stress (Takotsubo) cardiomyopathy. JAMA.

[7] Naruse Y, Sato A, Kasahara K (2011). The clinical impact of late gadolinium enhancement in Takotsubo cardiomyopathy: serial analysis of cardiovascular magnetic resonance images. J Cardiovasc Magn Reson.

[8] Sharkey SW, Windenburg DC, Lesser JR (2010). Natural history and expansive clinical profile of stress (tako-tsubo) cardiomyopathy. J Am Coll Cardiol.

[9] Gianni M, Dentali F, Grandi AM (2006). Apical ballooning syndrome or takotsubo cardiomyopathy: A systematic review. Eur Heart J.

[10] Akashi YJ, Nef HM, Lyon AR (2015). Epidemiology and pathophysiology of Takotsubo syndrome. Nat Rev Cardiol.

[11] Y-Hassan S, Tornvall P (2018). Epidemiology, pathogenesis, and management of takotsubo syndrome. Clin Auton Res.

[12] Scantlebury DC, Prasad A (2014). Diagnosis of Takotsubo cardiomyopathy. Circ J.

[13] Elesber AA, Prasad A, Lennon RJ, Wright RS, Lerman A, Rihal CS (2007). Four-year recurrence rate and prognosis of the apical ballooning syndrome. J Am Coll Cardiol.

